# Impact of an Artificial Intelligence–Powered Clinical Decision Support System for Acute Kidney Injury Prevention in the Intensive Care Unit: Single-Center Uncontrolled Before-and-After Implementation Study

**DOI:** 10.2196/87738

**Published:** 2026-07-08

**Authors:** Francesca Alfieri, Andrea Ancona, Alessandro Bacci, Simone Zappalà, Jordi Morillas Perez

**Affiliations:** 1 U-Care Medical S.r.l., Corso Castelfidardo 30/A, Torino, TO, 10129, Italy; 2 Servicio de Medicina Intensiva, Hospital de Barcelona (SCIAS), Barcelona, Spain

**Keywords:** acute kidney injury, artificial intelligence, clinical decision support systems, intensive care units, Kidney Disease: Improving Global Outcomes, KDIGO guidelines, implementation study, machine learning, renal function monitoring

## Abstract

**Background:**

Acute kidney injury (AKI) is a frequent and serious complication among hospitalized patients, particularly in critical care settings, where its incidence can exceed 50%. AKI is associated with increased mortality, prolonged hospitalization, dialysis dependence, and higher health care costs. Although the KDIGO (Kidney Disease: Improving Global Outcomes) guidelines emphasize supportive care, hemodynamic optimization, and avoidance of nephrotoxins, their implementation remains inconsistent, partly due to the lack of timely risk stratification. Recent advances in artificial intelligence have enhanced early prediction and detection of AKI, offering new opportunities to improve patient outcomes and intensive care unit (ICU) efficiency. The U-Care Renal Platform (UCRP; U-Care Medical S.r.l), a Conformité Européenne (CE)–marked artificial intelligence–powered medical device, integrates directly with the ICU electronic health record to continuously analyze patient data and predict the risk of moderate or severe AKI within 24 hours, providing actionable, guideline-based recommendations. While the predictive performance of UCRP has been validated previously, its real-world impact on clinical and operational outcomes in the ICU remains underexplored.

**Objective:**

This single-center uncontrolled before-and-after implementation study aims to evaluate the association between UCRP implementation and selected ICU clinical and operational outcomes in routine practice at SCIAS Hospital, Barcelona.

**Methods:**

This study was conducted as a retrospective service evaluation of a workflow-embedded clinical decision support system between March 2023 and March 2025. It included 202 postsurgical adult ICU patients. Outcomes of interest were assessed by comparing preimplementation and postimplementation periods. Months during which the UCRP was inactive were excluded from the analysis (total excluded duration: 10 months; 5 in the preimplementation period and 5 in the postimplementation period). The outcomes included the incidence of moderate-to-severe AKI (KDIGO stages 2 and 3), the use of nephrotoxic medications, the frequency of hypotensive episodes among patients with AKI, and the ICU length of stay.

**Results:**

During the postimplementation period, lower rates of moderate-to-severe AKI (9/99, 9.1% vs 12/103, 11.7%), nephrotoxic drug administration, and hypotensive episodes among patients with AKI were observed compared with the preimplementation period.

**Conclusions:**

Integration of the UCRP into ICU workflows was associated with differences in selected AKI-related process and intermediate clinical outcomes in this single-center uncontrolled before-and-after implementation study. However, given the study design, causal relationships cannot be established, and the findings should be interpreted as preliminary signals requiring confirmation in larger, controlled, and multicenter studies, including patient-centered outcomes.

## Introduction

Acute kidney injury (AKI) is a frequent and serious complication among hospitalized patients, particularly in critical care settings, where its incidence can exceed 50%. It is associated with a range of adverse outcomes, including increased risks of infections, chronic kidney disease, dialysis dependency, and heightened health care costs. Current management strategies emphasize supportive care, avoidance of nephrotoxic agents, optimization of hemodynamics, and renal replacement therapy when necessary [1]. However, despite the well-established KDIGO (Kidney Disease: Improving Global Outcomes) guidelines for AKI management, their inconsistent application in clinical practice results in variable patient outcomes.

Several studies have demonstrated that combining risk assessments for moderate-to-severe AKI with adherence to KDIGO guidelines can prevent AKI and promote its reversibility [2-4]. The 2012 KDIGO guidelines recommend an “AKI care bundle” approach that includes hemodynamic optimization, nephrotoxin avoidance, and glycemic control, but determining the patients who may benefit most from these interventions is complex in routine clinical practice [5-7].

Recent advancements in artificial intelligence (AI)–based risk assessment models have led to the development of AI models capable of accurately predicting AKI onset in real time in the intensive care unit (ICU), potentially impacting patient outcomes, ICU efficiency, and overall health care costs. In particular, several AI models have been developed and clinically validated recently [8-11]. However, the translation of these models into real-world clinical practice has so far remained limited. This gap is largely due to multiple barriers, including the lack of standardized data integration with hospital electronic health records (EHRs) and patient data management systems, regulatory and ethical constraints for AI-based medical devices, and the challenges in demonstrating clinical use and cost-effectiveness in prospective settings.

To address these challenges and evaluate the real-world impact of an AI-based risk prediction model in critical care, this study assessed the clinical implementation of the U-Care Renal Platform (UCRP), a Conformité Européenne (CE)–marked AI-powered medical device designed to support the early AKI risk prediction and management in ICU patients. The study was conducted in the ICU of SCIAS Hospital (Barcelona, Spain) using a preimplementation and postimplementation design. The primary objective was to evaluate the clinical and operational benefits of integrating this AI-driven platform into routine practice, with the ultimate goal of improving adherence to kidney care guidelines and supporting process and intermediate clinical outcomes in ICU care.

## Methods

### Study Design and Population

This was a single-center uncontrolled before-and-after implementation study conducted as a retrospective service evaluation based on routinely collected clinical data. Data from both the preimplementation and postimplementation periods were retrospectively extracted from the IntelliSpace Critical Care and Anesthesia (ICCA; Philips) database after completion of the postimplementation observation period. The implementation of UCRP was not introduced for research purposes but as part of routine clinical workflow, and no study-specific protocol or prospective data collection plan was defined prior to deployment. This study was not prospectively registered, as it was conducted as a retrospective service evaluation based on routinely collected clinical data without prospective assignment to an intervention.

The study was conducted at SCIAS Hospital in Barcelona, Spain, from March 2023 to March 2025, with a total of 202 patients included in the final analysis. The hospital used the ICCA system, which automatically collected all patient care data, including laboratory results, vital signs, demographics, and clinical observations. Thanks to the bidirectional integration between the ICCA EHR system and the UCRP via the Health Level Seven (HL7) protocol, real-time data exchange was established, enabling the UCRP to acquire data and display its results within the ICCA system without altering the clinicians’ existing workflow.

The UCRP was implemented as a workflow-embedded clinical decision support tool that provided continuously available, nonblocking, and visually salient outputs, including risk predictions, AKI staging, and KDIGO-oriented recommendations within the ICCA interface. The system did not autonomously modify treatment orders or enforce protocolized clinical actions, and all clinical decisions remained under the responsibility of the treating physicians. However, the system was designed to provide actionable information intended to support and potentially influence clinical decision-making during routine care.

### Ethical Considerations

This study was conducted as a local implementation and service evaluation project based on the secondary use of routinely collected clinical data within standard ICU care. The UCRP was implemented as part of routine clinical workflow and was not introduced for research purposes. No study-specific protocol, prospective assignment, or protocol-mandated modification of clinical management was defined. According to applicable institutional and national regulations governing retrospective analyses of anonymized clinical data and quality improvement activities, including local institutional governance policies and European data protection regulations [12], this type of study does not require formal ethics committee approval. Specifically, this evaluation falls under the category of service evaluation or quality improvement activities as defined by local institutional policies and applicable data protection regulations. Therefore, no ethics committee approval, exemption decision, or waiver reference number was obtained. All patients provided general hospital consent authorizing the use of clinical data for research and quality improvement purposes, and all data were handled in accordance with applicable data protection regulations, including the General Data Protection Regulation (GDPR) [12]. This approach is consistent with standard definitions of service evaluation and quality improvement activities under European and institutional governance frameworks.

### Study Period and Data Extraction

The study was divided into 2 implementation phases. The preimplementation period included retrospective data from adult patients admitted between March 2023 and March 2024, whereas the postimplementation period included adult patients admitted between March 2024 and March 2025. Months during which the UCRP was inactive due to technical deployment, maintenance, or clinical decision were excluded from both preimplementation and postimplementation periods to ensure comparability. Specifically, the excluded months were July, August, November, and December 2023; January, July, August, November, and December 2024; and January 2025. In total, 10 months were excluded across both periods (5 in the preimplementation period and 5 in the postimplementation period). Exclusion of inactive months was applied at the period level, whereas all other exclusion criteria were applied at the patient level.

After the exclusion of inactive months, each study period corresponded to approximately 7 months of effective observation, with minor differences in the distribution of excluded months between periods. In terms of patient-level exposure, this corresponded to 103 patients in the preimplementation period and 99 patients in the postimplementation period, with comparable ICU length of stay distributions between cohorts. All data were extracted from the ICCA database after the completion of the postimplementation phase. The final study population consisted of 103 patients in the preimplementation cohort and 99 patients in the postimplementation cohort.

### Additional Definitions and Data Handling

Baseline kidney function was defined using the most recent serum creatinine value available prior to ICU admission, as recorded in the EHR. In cases where preadmission values were not available, baseline creatinine was approximated using the first available measurement during ICU stay.

Missing creatinine and urine output data were handled by relying on available measurements without imputation, with no statistical imputation methods applied, and AKI staging was performed only when sufficient data were present according to the KDIGO criteria. All ICU stays were included regardless of discharge status (eg, transfer, discharge, or death), and outcomes were assessed over the entire ICU stay without censoring.

### Intervention

During the postimplementation period, the UCRP was used routinely for all patients admitted to the ICU. UCRP outputs were made available within the ICCA interface and through a dedicated central monitoring workstation, allowing clinicians to access the system during routine clinical workflows. The system was primarily intended for ICU physicians responsible for patient management.

UCRP outputs were implemented as a nonblocking, visually salient clinical decision support mechanism. Within the ICCA interface, alerts were displayed as visual cues (eg, red highlighting in the patient view) when predefined clinical thresholds or criteria were exceeded. Alerts were visible both within the ICCA patient view and in the dedicated UCRP interface accessed via the central monitoring workstation.

When accessing the UCRP interface, alerts were further emphasized through dynamic visual signals. No mandatory pop-up notification or user acknowledgment was required. This design ensured that clinically relevant information was clearly visible while preserving clinician autonomy and avoiding workflow disruption. No formal study-specific clinician training program was delivered as part of the evaluation, and alert logic and thresholds remained unchanged during the analyzed postimplementation period. No concurrent AKI-specific implementation initiatives were formally introduced; however, other unmeasured changes in clinical practice cannot be excluded.

The platform continuously monitored patient data and generated alerts when predefined or clinical criteria were exceeded. Importantly, the system generates heterogeneous alerts across its modules, including not only AKI risk prediction alerts (AKIRA index) but also KDIGO-based clinical recommendations such as hypotension, hyperglycemia, and nephrotoxic drug exposure. As a result, overall alert activity reflects a broad set of workflow-embedded monitoring signals rather than a single event-specific alerting function.

UCRP is an AI-based software that is CE-certified as a medical device according to the MDR (2017/745) regulation [13] and is currently available on the market. It is designed to support the prevention and treatment of AKI in patients admitted to the ICU.

The UCRP consists of 3 modules:

- STAGING module: In accordance with international KDIGO guidelines, the AKI stage (0‐3) is determined hourly during hospitalization for each patient admitted to the ICU by analyzing urine output and creatinine data.
- AKIRA module: Based on the proprietary AKIRA machine learning algorithm, the system predicts the onset of a moderate or severe AKI episode (stage 2 or 3) within the next 24 hours in patients identified as stage 0 or 1 using the STAGING module. During the ICU stay, AKIRA analyzes the patient’s clinical data, including laboratory tests, physiological parameters, and demographics, on an hourly basis to calculate a personalized risk index for the development of moderate or severe AKI.
- KDIGO bundle module: This module supports ICU clinicians in managing patients at risk for AKI in accordance with international KDIGO guidelines. Specifically, it allows for automatic monitoring of prescribed nephrotoxic medications, with notification of potentially harmful active ingredients requiring discontinuation or reassessment; automatic monitoring of nephrotoxic drug dosages, with timely recommendations for adjustments based on current glomerular filtration rate values; and automatic monitoring of hypotension and hyperglycemia events, with timely alerts for proactive clinical management.

### Inclusion and Exclusion Criteria

Patients were excluded from analysis if they (1) lacked age or gender information and (2) had an AKI stage 2 or 3 within the first 24 hours of ICU admission, as defined by KDIGO 2012 diuresis and creatinine criteria.

To ensure homogeneity between the preimplementation and postimplementation patient groups, only postsurgical patients admitted to the ICU were included in the study. Postsurgical patients were defined as those admitted to the ICU following a surgical procedure requiring postoperative monitoring, including elective and urgent major surgeries (eg, abdominal, vascular, and cardiothoracic procedures), as recorded in the hospital admission classification system.

Furthermore, patients with insufficient data to determine the AKI stage (such as at least 2 measurements of urine output and serum creatinine) were excluded. The proportion of patients excluded due to insufficient data for AKI staging is reported in the study flowchart given in the Results section for both study periods.

### End Points

The primary end point of the study was the incidence of moderate-to-severe AKI, defined according to KDIGO 2012 criteria as stage 2 or 3 AKI, based on both urine output and serum creatinine criteria during the ICU stay. Secondary end points included the occurrence of hypotensive and hyperglycemic episodes, the daily rate of nephrotoxic drug administration, and overall ICU efficiency metrics such as ICU length of stay. Moderate and/or severe AKI was classified according to KDIGO 2012 criteria. Hypotension was defined as a mean arterial pressure below 65 mm Hg, while hyperglycemia was defined as a blood glucose concentration exceeding 150 mg/dL, which is the upper limit of the ICU care bundle target range.

Drugs considered nephrotoxic included aminoglycosides (eg, amikacin, gentamicin), vancomycin, nonsteroidal anti-inflammatory drugs (eg, dexketoprofen, metamizole, acetylsalicylic acid, diclofenac), β-lactam antibiotics (eg, amoxicillin-clavulanate, ceftriaxone, cefazolin, ceftazidime, piperacillin-tazobactam, ampicillin, penicillin G), fluoroquinolones (eg, ciprofloxacin, levofloxacin), antiviral agents (eg, intravenous acyclovir), angiotensin-converting enzyme inhibitors (eg, captopril, enalapril), angiotensin II receptor blockers (eg, losartan, valsartan), diuretics (eg, furosemide, hydrochlorothiazide, mannitol), and trimethoprim-sulfamethoxazole.

The nephrotoxic drug administration rate per day was calculated by dividing the total number of nephrotoxic drug administrations for each patient by their ICU length of stay and normalizing this value to a 24-hour period. Sensitivity analyses were performed to assess the robustness of the findings. Specifically, analyses were repeated after excluding ICU stays shorter than 24 hours and by comparing early versus later months within the postimplementation period. Only the incidence of moderate-to-severe AKI was prespecified as the primary end point; secondary outcomes were considered exploratory.

### Statistical Analysis

Due to the exploratory nature and limited sample size of this pilot study, analyses focused on estimation rather than hypothesis testing. No formal hypothesis testing was performed. Binary outcomes are reported as proportions with 95% CIs calculated using the Wilson method. Continuous variables are summarized using mean (SD) or median (IQR), as appropriate based on the distribution of each variable.

## Results

### Baseline Characteristics of ICU Cohorts

**Figure 1.**
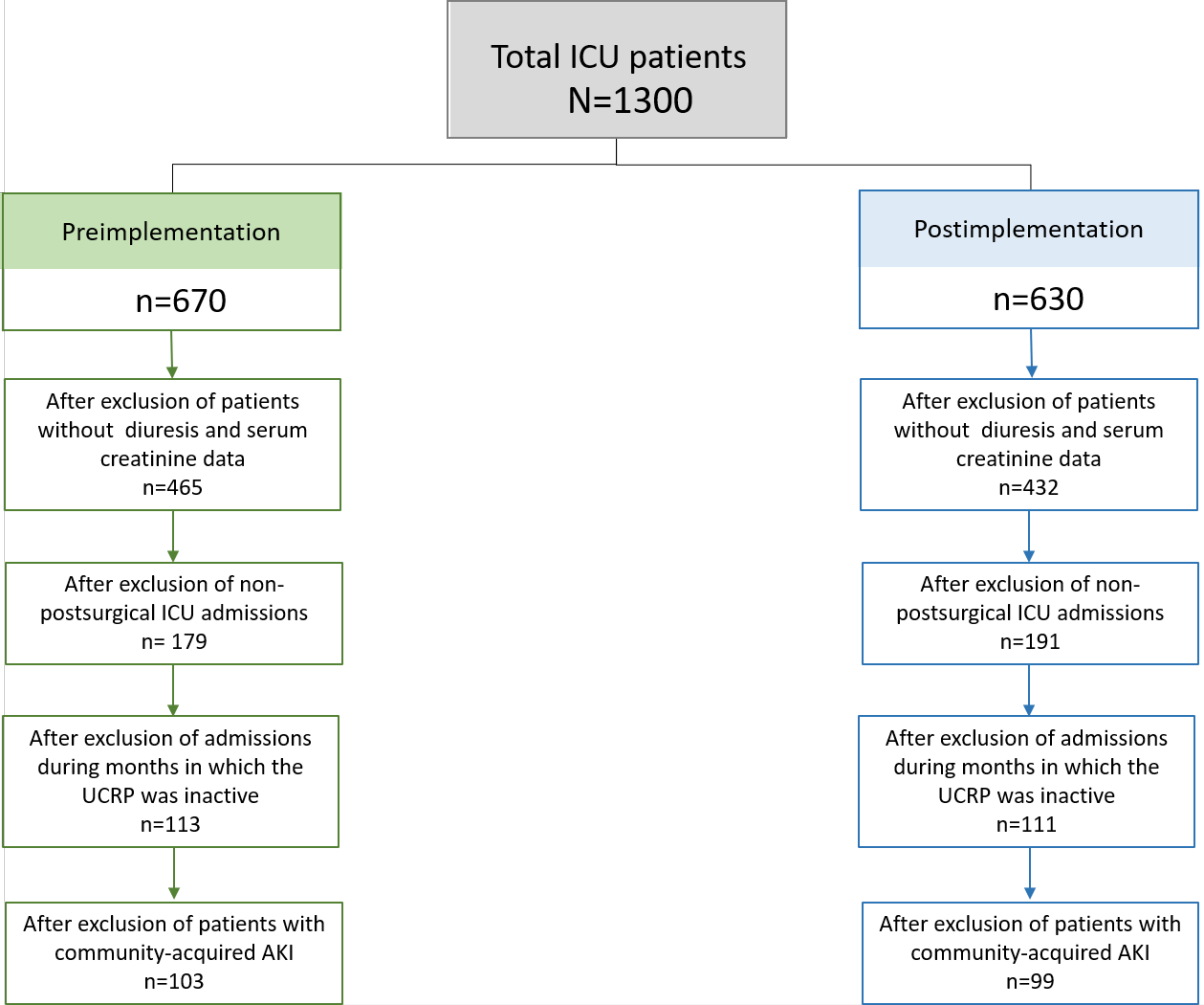
Flowchart of patient selection for the preimplementation and postimplementation cohorts. Sequential exclusions were applied for missing data, non-postsurgical admissions, inactive UCRP periods, and early AKI, resulting in final cohorts of 103 and 99 patients, respectively. AKI: acute kidney injury; ICU: intensive care unit; URCP: U-Care Renal Platform.

A total of 202 patients admitted to the ICU for postsurgical reasons were enrolled in this single-center uncontrolled before-and-after implementation study. In the final analysis, 103 were included in the preimplementation and 99 in the postimplementation cohorts (Figure 1). The study focused on surgical ICU patients, and the characteristics of the final patient population are summarized in Table 1. This table compares the demographics between the control and intervention cohorts. Data are presented as follows: binary variables are reported as counts (percentage of admissions), and continuous variables as median (IQR) or mean (SD), as appropriate.

**Table 1.** Patient characteristics in pre-UCRP^a^ and post-UCRP implementation cohorts.

	All patients (N=202)	Preimplementation (n=103)	Postimplementation (n=99)
Male, n/N (%)	102/202 (51)	51/103 (50)	51/99 (51)
Age (y), median (IQR)	72 (64‐78)	71 (63‐77)	74 (65‐80)
Age (y), n/N (%)			
18‐39	5/202 (2.5)	4/103 (3.9)	1/99 (1.0)
40‐49	6/202 (3.0)	4/103 (3.9)	2/99 (2.0)
50‐59	23/202 (11.4)	8/103 (7.8)	15/99 (15.1)
60‐69	43/202 (21.3)	24/103 (23.3)	19/99 (19.2)
70‐79	85/202 (42.1)	48/103 (46.6)	37/99 (37.4)
80+	40/202 (19.8)	15/103 (14.5)	25/99 (25.3)
Baseline serum creatinine (mg/dL), median (IQR)	0.95 (0.76‐0.98)	0.90 (0.76‐0.98)	0.95 (0.75‐0.98)
ICU^b^ length of stay (h), median (IQR)	22.5 (18.1‐45.1)	22.2 (18.4‐57.3)	23.8 (17.8‐42.7)

a UCRP: U-Care Renal Platform.

b ICU: intensive care unit.

The 2 cohorts were similar in terms of gender distribution and serum creatinine levels. However, patients in the intervention phase were slightly older (74 vs 71 y), and ICU length of stay was comparable between groups (median 23.8, IQR 17.8‐42.7 h vs 22.2, IQR 18.4‐57.3 h).

**Figure 2.**
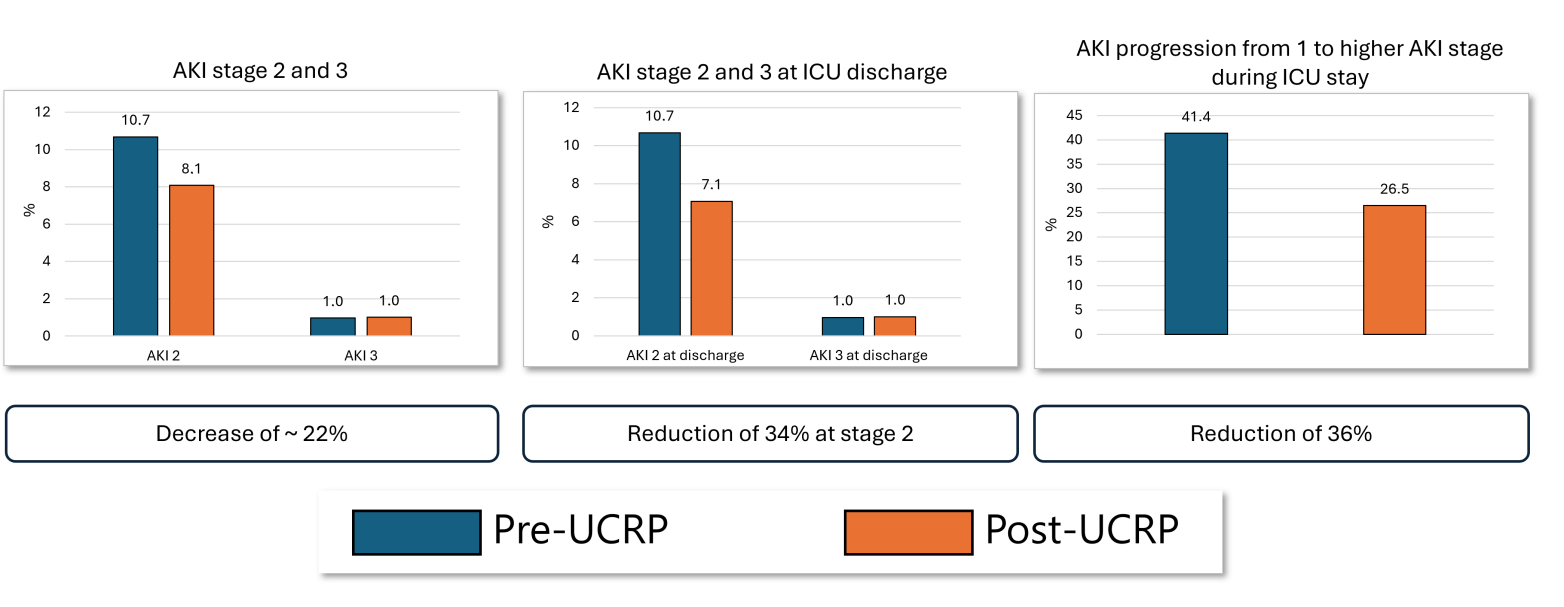
Comparison of AKI stage 2 and 3 incidence over the preimplementation and postimplementation study cohorts. AKI: acute kidney injury; ICU: intensive care unit; UCRP: U-Care Renal Platform.

The primary end point of this study was the incidence of moderate and severe AKI following the implementation of the UCRP. As shown in Figure 2A, the overall incidence of stage 2 and 3 AKI acquired during ICU stay decreased from 12 out of 103 (11.7%, 95% CI 6.8%‐19.3%) patients to 9 out of 99 (9.1%, 95% CI 4.9%‐16.4%) patients, corresponding to an absolute difference of −2.6 percentage points and a relative reduction of 22%. Figure 2B shows a similar trend at ICU discharge, with stage 2 AKI decreased from 11 out of 103 (10.7%, 95% CI 6.1%‐18.1%) patients to 7 out of 99 (7.1%, 95% CI 3.5%‐13.9%) patients, corresponding to an absolute difference of −3.6 percentage points and a relative reduction of 34%, while stage 3 AKI remained unchanged compared to the preimplementation period (pre: 1/103, 1.0%, 95% CI 0.2%‐5.3%; post: 1/99, 1.0%, 95% CI 0.2%‐5.5%). The proportion of patients progressing from AKI stage 1 to stage 2 or stage 3 during ICU stay decreased from 12 out of 29 (41.4%, 95% CI 25.5%‐59.3%) patients to 9 out of 34 (26.5%, 95% CI 14.6%‐43.1%) patients, corresponding to an absolute difference of −14.9 percentage points and a relative reduction of 36% (Figure 2C).

Furthermore, among patients who experienced AKI of any stage during their ICU stay, a 28% relative reduction in the total number of daily administrations of nephrotoxic drugs was observed, with the median number of daily nephrotoxic drug administrations decreasing from 9 (IQR 4‐16) to 6.5 (IQR 3‐10) after the intervention.

To provide contextual information on system activity during the postimplementation period, we assessed the occurrence of AKI risk alerts (AKIRA index) during ICU stay. During the postimplementation period, 16.2% (16/99) of patients generated at least 1 AKI risk alert. As AKIRA is designed to identify patients at increased risk of developing moderate-to-severe AKI, this metric reflects detection of at-risk patients rather than confirmed AKI stage 2 or 3 events. Accordingly, it should be interpreted as a measure of risk identification within the clinical workflow rather than as an indicator of model performance.

The last analysis performed concerns the number of hypotension episodes developed by patients with AKI of any stage during ICU stay. The percentage of patients who developed hypotension episodes decreased from 11 out of 29 patients (37.9%, 95% CI 22.7%‐56.0%) in the preimplementation cohort to 9 out of 34 patients (26.5%, 95% CI 14.6%‐43.1%) in the postimplementation cohort, corresponding to an absolute difference of −11.4 percentage points and a relative reduction of 30%.

Sensitivity analyses showed consistent trends across specifications, with no substantial deviation from the primary analysis.

### Illustrative Clinical Workflow Case

**Figure 3.**
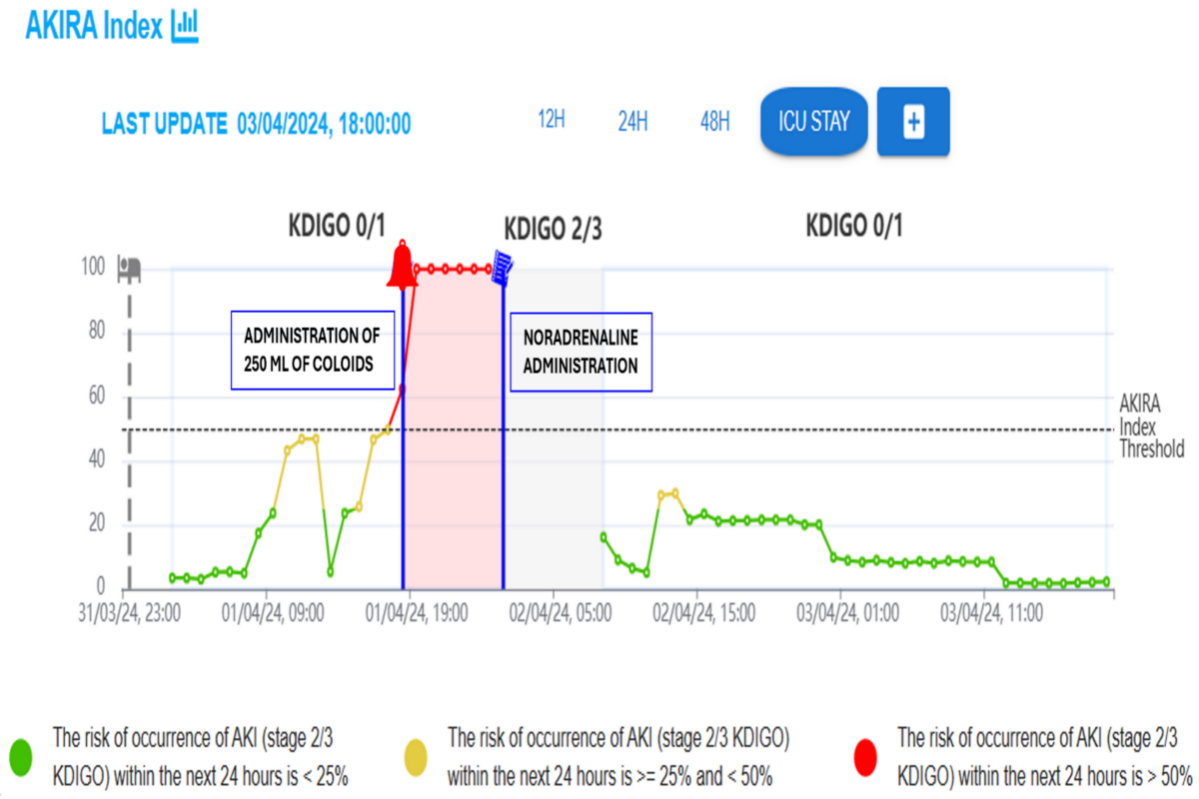
Illustrative case temporally associated with UCRP alert generation and subsequent clinical actions. AKI: acute kidney injury; KDIGO: Kidney Disease: Improving Global Outcomes; UCRP: U-Care Renal Platform.

To illustrate a real-world clinical workflow example temporally associated with UCRP alert generation, Figure 3 presents the trend of the AKIRA index during an ICU hospitalization. The patient was an 81-year-old male with no prior history of chronic kidney disease. After 19 hours of ICU admission, the AKIRA index exceeded the alert threshold, indicating a >50% probability of developing AKI stage 2 or 3 within the following 24 hours.

According to retrospective review, the alert was temporally associated with clinical interventions. Specifically, the patient received 2 interventions, administration of colloids and initiation of noradrenaline, in the context of hypotension and suspected prerenal AKI.

Despite transient progression to AKI stage 2 or 3, the AKI episode resolved within 24 hours. The temporal sequence of alert generation and clinical interventions is presented descriptively. While these observations are compatible with the hypothesis that alert-informed clinical actions may influence the subsequent clinical course, no causal relationship can be established based on this single illustrative case.

The patient’s ICU stay was brief (<3 d) and was discharged without an AKI diagnosis.

## Discussion

### Clinical Impact, Interpretation, and Translational Evaluation of the UCRP

This pilot single-center uncontrolled before-and-after implementation study assessed the impact of a commercially available clinical decision support system (CDSS), the UCRP, which integrates AI model outputs and guideline-based suggestions for early prediction and management of AKI stage 2 or 3 in postsurgical ICU patients over a 14-month period.

In a similar single-center uncontrolled before-and-after implementation study where only guideline-based suggestions for AKI and automatic AKI detection were implemented [3], it was demonstrated that the overall incidence of AKI was reduced by approximately 16% in the intervention cohort (11% for stage 1, 14% for stage 2, and 28% for stage 3), compared with our measured reductions of 25% for AKI stages 1 to 3 and 35% for AKI stages 2 and 3. Preliminary findings from this study indicate that implementation of a system integrating real-time AKI prediction within 24 hours and data-driven KDIGO Bundle recommendations was associated with differences in selected process and intermediate clinical outcomes and ICU operational metrics. More broadly, evidence demonstrating improvement in patient-centered outcomes for AI-based CDSSs remains limited, and rigorous real-world evaluation frameworks are increasingly recommended [14-16].

A multicenter study from Al Jaghbeer et al [17] demonstrated that implementation of a CDSS for AKI resulted in a modest but sustained decrease in hospital mortality, dialysis use, and length of stay. In contrast, our preliminary results indicate a reduction in the incidence of AKI, but we did not observe a decrease in mortality or dialysis use. This discrepancy may be attributed to differences in study design, patient populations, as well as clinical and institutional practices.

Our study, conducted in a postsurgical ICU with a specific patient population, showed associations between the postimplementation period and lower rates of AKI as well as favorable differences in selected management-related outcomes. Only a subset of patients (16/99, 16.2%) generated AKI risk alerts, indicating that the system selectively identifies patients at increased risk rather than broadly triggering alerts across the entire cohort.

Although the study design limits causal inference, these findings may be consistent with the intended role of the platform within the clinical workflow, namely, the availability of visually highlighted, nonblocking alerts, real-time AKI stage calculation, and KDIGO-oriented recommendations. By providing clinicians with timely and actionable information, including alerts and recommendations based on real-time data, the software was intended to support clinical decision-making in patients at risk of AKI. The implementation of the software’s guidance on nephrotoxic drug management may be associated with the observed lower rates. This pattern may be consistent with multiple potential mechanisms. First, the software’s predictive model may enable earlier identification of patients at risk of AKI, which may be associated with differences in clinical decision-making regarding treatment adjustments, such as reducing dosages, discontinuing nephrotoxic agents, or selecting safer therapeutic alternatives, thereby contributing to reduced exposure rather than implying a direct causal effect. Second, the observed lower AKI incidence may also be associated with a reduced need for nephrotoxic medications, as AKI frequently requires treatments involving potentially nephrotoxic antibiotics or contrast media.

Notably, nephrotoxic drugs are well-established risk factors for AKI development; thus, their reduced use may be associated with the observed patterns. Overall, these findings are consistent with safer prescribing patterns during the postimplementation period, although no causal relationship can be established.

These implementation characteristics provide context for interpreting the observed associations and support the reproducibility of the system in clinical practice. These findings should be interpreted as hypothesis-generating and warrant confirmation in controlled and multicenter prospective studies.

From a translational research perspective, this study can be considered an early-stage clinical evaluation of an AI-based CDSS, consistent with the framework proposed by the Developmental and Exploratory Clinical Investigation of Decision Support Systems Driven by Artificial Intelligence guidelines for the staged assessment of AI-driven interventions in health care [18]. In line with evidence-based medicine principles, the clinical evaluation of AI-based software as a medical device is generally expected to follow a progressive pathway ranging from retrospective and prospective observational studies to nonrandomized controlled trials and, ultimately, multicenter randomized controlled trials aimed at generating higher-certainty evidence and supporting Grading of Recommendations Assessment, Development, and Evaluation–based recommendations [19,20]. In this context, future research will focus on extending the evaluation of the UCRP across multiple centers and patient populations, with the inclusion of more structured and controlled study designs. Recent examples of AI-based systems following this trajectory, such as Park et al [21], further highlight the importance of structured validation pathways in demonstrating clinical effectiveness in real-world settings.

### Limitations and Conclusions

A key limitation of this study is the relatively small sample size, as the analysis was conducted in a single ICU department and limited to surgical patients admitted over a 14-month period. This narrow scope may affect the generalizability of the findings. Although CIs were estimated for binary outcomes, the limited sample size results in wide uncertainty ranges and limited statistical power; therefore, findings should be interpreted cautiously.

Because most authors are employees of the manufacturer of the evaluated system, the possibility of sponsor-related bias in analysis and interpretation should also be considered. In addition, this was an uncontrolled before-and-after implementation study; therefore, the certainty of causal inference is limited, as such designs are generally considered to provide low to very low certainty for causal inference because alternative explanations for observed differences cannot be excluded.

Observed differences between the preimplementation and postimplementation periods may be influenced by secular trends, regression to the mean, case-mix variations, or other concurrent changes in clinical practice. Furthermore, detailed implementation metrics, such as alerts per patient-day, acknowledgment rates, or clinician interaction patterns, were not available due to system architecture, limiting the ability to fully characterize system uptake and its relationship with clinical outcomes.

Standardized severity scores and comorbidity indices (eg, Sequential Organ Failure Assessment, Acute Physiology and Chronic Health Evaluation II, Simplified Acute Physiology Score II, Charlson Comorbidity Index) were not consistently available and therefore could not be included in the analysis. This limits adjustment for case-mix differences between periods and increases the possibility of residual confounding.

To address these limitations, future work will extend the evaluation to a larger and more heterogeneous patient population across multiple hospitals and ICU specialties, allowing for a more comprehensive and statistically robust assessment of the system’s effectiveness. Moreover, the exclusive focus on postsurgical ICU patients further limits the applicability of the results to other clinical contexts.

Furthermore, this study did not include follow-up analyses of long-term renal or patient-centered outcomes, such as major adverse kidney events, dialysis dependence, or mortality. Future prospective evaluations incorporating these end points will be essential to fully characterize the sustained clinical impact of the system.

The absence of a formal ethics committee reference number may limit the external interpretability of the study governance framework.

## Abbreviations

AI: artificial intelligence
AKI: acute kidney injury
CDSS: clinical decision support system
CE: Conformité Européenne
EHR: electronic health record
GDPR: General Data Protection Regulation
ICCA: IntelliSpace Critical Care and Anesthesia
ICU: intensive care unit
KDIGO: Kidney Disease: Improving Global Outcomes
UCRP: U-Care Renal Platform
